# Inhibition of PD-L1/PD-1 Checkpoint Increases NK Cell-Mediated Killing of Melanoma Cells in the Presence of Interferon-Beta

**DOI:** 10.3390/cancers17243899

**Published:** 2025-12-05

**Authors:** Anna Makowska, Lian Shen, Christina Nothbaum, Diana Panayotova-Dimitrova, Maria Feoktistova, Amir S. Yazdi, Udo Kontny

**Affiliations:** 1 Division of Pediatric Hematology, Oncology and Stem Cell Transplantation, Medical Faculty, RWTH Aachen University, 52074 Aachen, Germany; lshen@ukaachen.de (L.S.); christina.nothbaum@rwth-aachen.de (C.N.); ukontny@ukaachen.de (U.K.); 2 Department of Dermatology and Allergology, University Hospital RWTH Aachen, Pauwelsstraße 30, 52074 Aachen, Germany; ddimitrova@ukaachen.de (D.P.-D.); mfeoktistova@ukaachen.de (M.F.); ayazdi@ukaachen.de (A.S.Y.); 3 Center for Integrated Oncology Aachen Bonn Cologne Duesseldorf (CIO ABCD), 52074 Aachen, Germany

**Keywords:** melanoma, interferon-β, natural killer cells, PD-1, PD-L1

## Abstract

**Background/Objectives:** Melanoma is a highly malignant skin tumor with poor response to conventional chemotherapeutic regimens. Melanoma cells induce cytotoxic T cell-mediated immune responses, and immunotherapy has significantly improved survival rates for patients with advanced disease. **Methods**: Here, we investigate NK cell-mediated melanoma cell killing and its regulation by PD-L1/PD-1 blockade and IFNβ. Four melanoma cell lines were used in this study. To evaluate NK cell cytotoxicity, cells were exposed to NK cells with or without IFNβ. The calcein release assay was used to measure cell death, while specific inhibitors and siRNA silencing were applied to determine the contribution of individual effector pathways. **Results**: NK cells were able to kill melanoma cells with sensitivity to killing varying between different cell lines. Cytotoxic effects were mainly mediated through activation of the TRAIL signaling cascade. In cell lines with low sensitivity to NK cell killing, expression of PD-L1 was noted and killing by NK cells could be significantly increased by inhibition of the PD-L1/PD-1 checkpoint. Killing of melanoma cells could be further increased by incubation of NK cells with IFNβ. **Conclusions**: Our results point to a role of NK cells in the killing of melanoma cells and a potential clinical benefit of a combination therapy of IFNβ and anti-PD-1 antibody.

## Simple Summary

Melanoma is an aggressive cutaneous malignancy with limited responsiveness to conventional chemotherapy. While immunotherapeutic strategies, particularly immune checkpoint inhibitors, have markedly improved outcomes in advanced-stage disease, therapeutic resistance remains a challenge. This study investigates the cytotoxic potential of natural killer (NK) cells against melanoma cell lines and evaluates the modulatory effects of interferon-beta (IFNβ) and PD-L1/PD-1 immune checkpoint blockade. Four melanoma cell lines were assessed for NK cell-mediated lysis using a calcein release assay. Cytotoxicity varied among cell lines and was predominantly mediated via the TRAIL (TNF-related apoptosis-inducing ligand) pathway. In cell lines exhibiting reduced NK cell sensitivity, elevated PD-L1 expression was observed. Blockade of the PD-L1/PD-1 axis significantly enhanced NK cell cytotoxicity, an effect further potentiated by IFNβ stimulation. These findings underscore the therapeutic potential of combining IFNβ with PD-1 inhibitors to augment NK cell-mediated antitumor responses in melanoma, offering a rationale for combinatorial immunotherapeutic approaches in clinical settings.

## 1. Introduction

More than 300,000 new cases of melanoma were estimated worldwide in 2020 [1]. Early diagnosis of melanoma is crucial, as it can lead to a survival rate of up to 99% of patients [2,3]. In contrast, advanced melanoma has a poor prognosis and its incidence rate continues to increase in many countries [4,5]. Postoperative adjuvant therapy in higher stages is important in order to prevent tumor recurrence and metastasis [6]. Various modalities, including immune therapy, chemotherapy, and radiation, have been investigated as adjuvant therapies; although the efficacy of advanced melanoma treatment has improved in recent years, it still remains unsatisfactory [3,7].

It was demonstrated that the ability of melanoma to induce an immune response contributes to patient survival. Melanoma induces cytotoxic T cell-mediated immune responses, and tumor-infiltrating lymphocytes are associated with spontaneous tumor regression and a favorable prognosis in primary melanoma [8,9]. High immunogenicity of melanoma and spontaneous remissions encouraged clinical trials in melanoma patients with immunostimulatory regimens such as interleukin-2 (IL-2). The adoptive transfer of ex vivo-expanded, IL-2-activated, tumor-infiltrating T cells resulted in an objective response in 50% of treated melanoma patients [10]. Unfortunately, T-cell- and cytokine-based strategies are limited by efficacy and side effects, making the development of more efficient approaches necessary. Unlike T cells, NK cells are activated, amongst other mechanisms, by the downregulation of HLA class I molecules, thereby overcoming the hurdle of tumor immune escape [11]. Previous experiments demonstrated that NK cells can recognize and destroy melanoma cells in vitro [12]. The activity of NK cells against melanoma in vivo was demonstrated in murine models [13,14]. Intravenous NK cell therapy in mice with melanoma decreased tumor size by 40–90% and prolonged survival 1.5–2.5-fold [15]. In patients with melanoma, the infusion of “activated” autologous or allogeneic NK cells showed encouraging results in early clinical trials [16].

The efficiency of NK cells depends on the balance between inhibitory and activating signals mediated through NK surface receptors upon contact with their ligands. Impairment of NK cell function via various immune checkpoint molecules is observed in melanoma patients [17,18]. Immune checkpoint inhibitor therapies are highly effective in patients with advanced melanoma. Treatment with the anti-PD-1 monoclonal antibody nivolumab led to durable responses in 30% to 40% of patients [19]. Even higher rates of activity, with a five-year survival rate of 52%, were observed when nivolumab was combined with the anti-cytotoxic T-lymphocyte-associated antigen 4 (CTLA) antibody ipilimumab [20]. Nivolumab is licensed as an adjuvant treatment in metastasized melanoma. Type I interferons are also used as an adjuvant therapy for the treatment of melanoma and demonstrate direct and indirect antitumor effects [21,22]. In melanoma patients, peritumoral injection of IFNβ recruits and stimulates immune effector cells, such as cytotoxic T cells, into the tumor microenvironment [23], suggesting a possible mechanism for the therapeutic effect of IFNβ in the treatment of melanoma. As type I interferons induce the expression of PD-L1 on tumor cells and PD-1 on immune cells [24], there is a rationale to combine treatment with PD-1 inhibition and type I interferon in melanoma. In a phase II trial, the combination of the anti-PD-1 inhibitor pembrolizumab with PEG-IFNα exhibited significant clinical activity in patients with melanoma [24]. As it has been demonstrated that not only T cells can be activated by IFNβ but also NK cell cytotoxicity [23,25], our analysis focused on the cytotoxic activity of NK cells against melanoma cells and how this process may be modulated by IFNβ and PD-L1/PD-1 checkpoint blockade.

## 2. Materials and Methods

### 2.1. Cell Lines and Culture

We used a panel of four human primary melanoma cell lines representing vertical growth phase (VGP) disease—the invasive stage characterized by vertical dermal expansion—but differing in driver genotype and aggressiveness: WM-115, WM-278, WM-1366, and WM-3248. All four lines are derived from primary cutaneous melanomas and are tumorigenic with competence for metastasis in vivo. WM-115 and WM-278 are considered less invasive VGP primary melanomas, whereas WM1366 and particularly WM-3248 display a more invasive VGP phenotype with higher basal invasion and oxidative phosphorylation. WM-115, WM-3248, and WM-278 have an activating BRAF mutation; WM-1366 carries an NRAS mutation [26]. The differences in genotype and invasiveness among the cell lines are expected to influence their susceptibility to NK cell-mediated killing and PD-1/PD-L1 blockade. Highly invasive VGP lines (WM-1366 and WM-3248) may exhibit greater resistance due to enhanced survival signaling and metabolic adaptation, whereas less invasive BRAF-mutant lines (WM-115 and WM-278) could be more sensitive. These variations are relevant for interpreting heterogeneity in killing assays. We obtained the cells as follows: WM-115 from the ATCC (Manassas, VA, USA); WM-3248 and WM-278 from Rockland Immunochemicals Inc. (Limerick, PA, USA); and WM-1366 from the Wistar Institute (Philadelphia, PA, USA). Cells were cultured in RPMI1640 (Gibco, Paisley, UK) medium supplemented with 10% fetal bovine serum (FCS) (Gibco, Paisley, UK) and 100 U/mL penicillin and 100 mg/mL streptomycin (Gibco, NY, USA). Cell lines used in this study were tested regularly to be free of mycoplasma and were authenticated through STR profiles.

### 2.2. Isolation of Primary Human NK Cells

NK cells were isolated from PBMCs of four healthy donors via Ficoll-Hypaque density gradient centrifugation. Buffy coat use was consented. CD56^+^ cells were magnetically selected (Miltenyi, Bergisch Gladbach, Germany), yielding 94% purity as verified by flow cytometry (anti-CD56-PE- clone: NCAM-1, # 555516; anti-CD3-APC-H7-clone: SK7, # 560275; BD-Biosciences, Franklin Lakes, NJ, USA). CD3 contamination in purified NK cells was less than 2%. Purified NK cells were cultivated in RPMI1640 medium (Gibco, Paisley, UK) supplemented with 10% FCS (Gibco, Paisley, UK) and 100 U/mL penicillin and 100 mg/mL streptomycin (Gibco, NY, USA).

### 2.3. Reagents

Human recombinant interferon-beta (IFNβ) was obtained from R&D System (New York, NY, USA, #8499-IF-010) and soluble, recombinant human TRAIL from Enzo Life Science (Paris, France). Antibodies used for flow cytometry, anti-TRAIL (clone 2E5, #ALX-804-296-C100), anti-TRAIL-R1 (clone TR1.02, #ALX-804-665-C100), and anti-TRAIL-R2 (clone DJR2-2, #ALX-804-913-C100) were from Enzo Life Science (Paris, France). Anti-FAS (clone ZB4, #05-338) was from MerckMillipore (Darmstadt, Germany), Con A from Sigma (St. Louis, MO, USA, #C5275) and anti-human PD-L1 (clone 130021) from R&D Systems (Minneapolis, MN, USA, #MAB1561). Isotype controls, IgG-APC (clone MOPC-21, #400122) and IgG-PE (clone MOPC-21#400112) and anti-human Ki-67 (clone Ki 67, #350514), were from BioLegend (San Diego, CA, USA). Nivolumab was purchased from Bristol-Myers (Anagni, Italy, PZN: 11024618). PD-L1 knockdown employed siRNA (Dharmacon, Freiburg, Germany, #L-015836-01-0010) with scrambled RNA controls (Dharmacon, Freiburg, Germany, #D-001810-20-0005). Calcein-AM was purchased from Thermo Fisher (Eugene, OR, USA, #C3100MP). PE Active Caspase-3 Apoptosis Kit (#550914) and human BD Fc Block was from BD Pharmingen (#564219, Franklin Lakes, NJ, USA). Antibodies used for immunoblots: anti–PD-L1 mAb (Cell Signaling, Danvers, MA, USA, #13684), anti–TRAIL-R1 (ProSci, Poway, CA, USA, #1139), anti–TRAIL-R2 (Sigma, St. Louis, MO, USA, #D-3938), β-Tubulin (Sigma, Darmstadt, Germany, #T4026), and goat anti-rabbit IgG (R&D Systems, Minneapolis, MN, USA, #BAF017).

### 2.4. Flow Cytometry

Melanoma and NK cells, pretreated or not with 1000 U/mL of IFNβ for the indicated time periods, were suspended at a density of 1 × 10^6^ cells in 500 μL of medium. Surface TRAIL expression was analyzed by incubating NK cells with anti-TRAIL antibody (5 µL). The expression of TRAIL receptor 1 (TRAIL-R1) and TRAIL receptor 2 (TRAIL-R2) on melanoma cells was assessed by incubation with anti-TRAIL-R1 (5 µL) or anti-TRAIL-R2 (5 µL) for 1 h on ice. Isotype controls were included. Following PBS washes, an APC-conjugated goat anti-mouse secondary antibody (1:200) was applied for 1 h. Checkpoint modulators were evaluated using anti-PD-1-PE (NK cells, 10 µL) and anti-PD-L1-PE (melanoma cells, 10 µL). Flow cytometry was performed using a BD FACS Canto II (BD Biosciences, Erembodegem, Belgium), and data were analyzed with FlowJo software 10.8.1 (FlowJo LLC, Ashland, OR, USA). All experiments were conducted in technical triplicates.

### 2.5. Analysis of Apoptotic and Proliferative Markers (Active Caspase-3 and Ki-67)

Intracellular levels of active caspase-3 and Ki-67 were quantified by incubating NK–melanoma co-cultures with anti-caspase-3-PE (5 µL) and anti-Ki-67-APC (5 µL). NK and melanoma cell populations were identified using anti-CD56-PE (5 µL) and anti-CD56-APC (5 µL). Samples were incubated for 30 min at 4 °C, washed, and subsequently analyzed by flow cytometry. A negative control sample incubated with APC-IgG or PE-IgG was processed in parallel. Each assay was performed in three independent experiments.

### 2.6. Calcein Release Assay

The calcein acetoxymethyl (calcein-AM) release assay was used to assess the effect of NK cell-induced cytotoxicity. The assay was performed as previously reported [24,27]. Briefly, melanoma cells were loaded with 15 µM calcein-AM (30 min, 37 °C) and co-incubated with NK cells at E:T ratios (50:1–1:1) for 4 h. Maximum release was induced with 4% Triton; spontaneous release was measured in melanoma-only controls. In all cytotoxicity assays, spontaneous lysis was subtracted from the total lysis values to ensure that the reported data reflected NK cell-mediated killing rather than baseline cell death. This correction is standard practice because melanoma cells exhibit a relatively high level of spontaneous lysis, which could otherwise confound interpretation of NK cell activity. The detailed results of spontaneous lysis for all melanoma cell lines are provided in Supplementary Figure S1. RFU values from supernatants were used to calculate specific lysis: [(RFU value in respective treatment − RFU value in control (spontaneous release))/(RFU value Triton (maximum release) − RFU value in control (spontaneous release)) × 100].

### 2.7. Analysis of NK Cell Cytotoxicity

Death ligand involvement was tested by pretreating NK cells with blocking anti-TRAIL mAb (clone 2E5, 100 ng/mL) and melanoma cells with anti-FAS mAb (clone ZB4, 100 ng/mL) for 1 h before co-culture. NK cells were also treated with Con A (2.5 µg/mL, 2 h) to block perforin/granzyme B or with nivolumab (1 h) to inhibit PD-1. Cytotoxicity was measured via calcein-AM release. To determine whether nivolumab induces NK cell activation through Fcγ receptor engagement rather than exclusively via PD-1 blockade, NK cells were pre-incubated with 25 µg/mL human BD Fc Receptor Blocker for 10 min prior to nivolumab treatment.

### 2.8. Transfection of siRNA

Melanoma cells were seeded at 10^5^ cells/well in 24-well plates. When cells had reached about 80% confluency, cells were PBS-washed and transfected with Lipofectamine (Invitrogen, Carlsbad, CA, USA, #11668019) using PD-L1 siRNA or scrambled siRNA. After 16 h, medium was replaced and cells treated with IFNβ (1000 U/mL, 24 h). Transfection efficiency was verified by PD-L1 expression (flow cytometry and immunoblotting). Triplicate experiments were conducted.

### 2.9. Immunoblot

Cells were rinsed with Dulbecco’s phosphate-buffered saline (DPBS) and subjected to lysis according to previously established protocols [28]. For protein extraction, a lysis buffer was prepared containing 30 mM Tris-HCl (pH 7.5), 120 mM NaCl, 10% (*v*/*v*) glycerol, 1% (*v*/*v*) Triton X-100, and Complete™ protease inhibitor tablets(two tablets per 100 mL; Roche, Basel, Switzerland). Cell lysates were incubated on ice for 20 min to ensure efficient solubilization of membrane-associated proteins, followed by clarification through centrifugation at 14,000× *g* for 10 min at 4 °C. Subsequently, 5 µg of total protein was resolved by SDS-PAGE on a 4–12% Bis-Tris gradient gel (NP0329BOX; Thermo Fisher Scientific, Waltham, MA, USA) after denaturation at 95 °C for 5 min in Laemmli buffer. Proteins were electrotransferred onto PVDF membranes (IB24001X3; Thermo Fisher Scientific) using a semi-dry transfer system. Membranes were blocked for 2 h at room temperature in TPBS supplemented with 5% (*w*/*v*) non-fat dry milk (70166-500G; Sigma-Aldrich, St. Louis, MO, USA) and subsequently washed with TPBS. Immunodetection was performed by overnight incubation at 4 °C with primary antibodies, followed by 1 h incubation at room temperature with HRP-conjugated secondary antibodies. Signal development was achieved using Immobilon^®^ Forte Western HRP substrate (WBLUF0500; Merck, Darmstadt, Germany).

### 2.10. Statistical Analysis

Data are represented as a mean ± S.E.M. For each calcein release assay, three independent experiments were performed using NK cells from three different donors, with each condition measured in quintuplicate. For flow cytometric analyses, three independent experiments were conducted, each in triplicate. Differences between groups were examined for significant differences by unpaired *t*-test. The level of statistical significance was set at *p* < 0.05.

## 3. Results

### 3.1. NK Cells Kill Melanoma Cells

**Figure 1:**
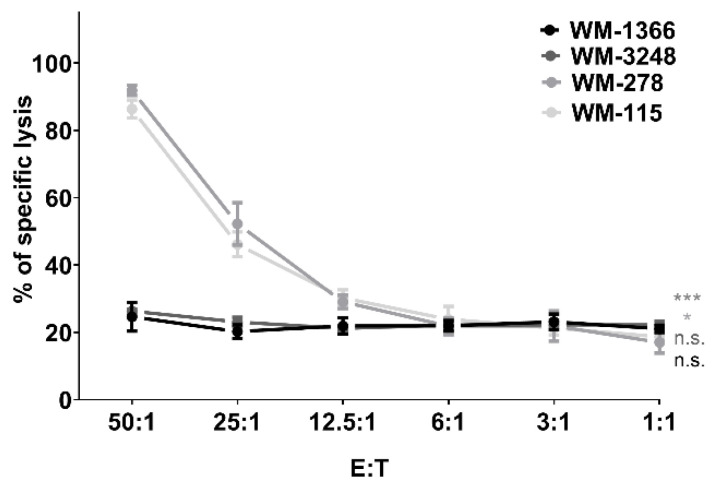
NK cell-mediated killing of melanoma cells. NK cells were co-cultured with four melanoma cell lines at an effector/target (E:T) ratio from 1:1 to 50:1. Triplicate cytotoxicity assays: NK cells (healthy donors) co-cultured with calcein-labeled targets (4 h). Calcein release in supernatants measured via SpectraMax microplate reader to determine lysis. Data are presented as means ± S.E.M. (Student’s *t*-test; * *p* < 0.05; *** *p* < 0.001, n.s. not significant).

First, we tested whether NK cells showed cytotoxic activity against melanoma cells. Melanoma cells, labeled with calcein, were incubated with NK cells at increasing effector /target (E:T) ratios (1:1–50:1) for 4 h. Supernatant calcein levels were measured to assess melanoma cell lysis. NK cells induced high levels of calcein release at a 50:1 E:T ratio in two melanoma cell lines: WM-278: 91.90 ± 1.51% and WM-115: 86.38 ± 2.67%. In contrast, a much lower calcein release was observed at the same E:T ratio for the two other melanoma cell lines: WM-1366: 24.61 ± 4.29% and WM-3248: 26.32 ± 0.68%. At the low effector /target ratio of 6:1, all melanoma cell lines demonstrated a similar sensitivity against NK cells from 21.30 ± 2.03% to 26.15 ± 2.35% (Figure 1). NK cells from three healthy donors showed similar cytotoxicity, confirming their variable ability to kill melanoma cells.

To further substantiate the cytotoxic activity of NK cells against melanoma cells, we analyzed markers of apoptosis and proliferation. Additional experiments were performed to assess the expression of active caspase-3 as an indicator of apoptosis and Ki-67 as a marker of proliferation. When NK cells are present in higher ratios, particularly with melanoma cells that are sensitive to NK activity, apoptosis increases as indicated by higher levels of active caspase-3, while proliferation decreases as reflected by lower expression of Ki-67 (Supplementary Figure S2).

### 3.2. NK Cells Kill Melanoma Cells via TRAIL

**Figure 2:**
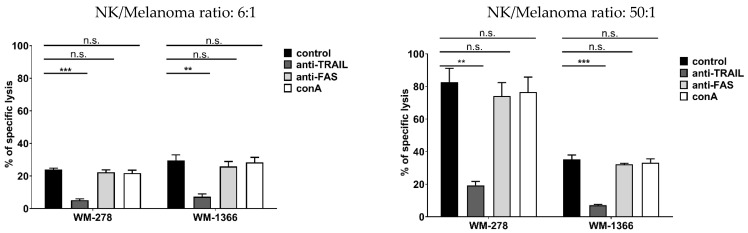
Cytotoxicity of NK cells is mediated by the TRAIL signaling pathway. NK cells were incubated with a blocking anti-TRAIL antibody or Con A before co-culture with melanoma cells, or melanoma cells were treated with the FAS-blocking mAb (ZB4) for 1 h before co-culture at an E:T ratio of 6:1 (**left**) or 50:1 (**right**). Melanoma lysis was then measured via calcein release assay. Data are presented as means ± S.E.M. (Student’s *t*-test; ** *p* < 0.01; *** *p* < 0.001, n.s. not significant).

**Figure 3:**
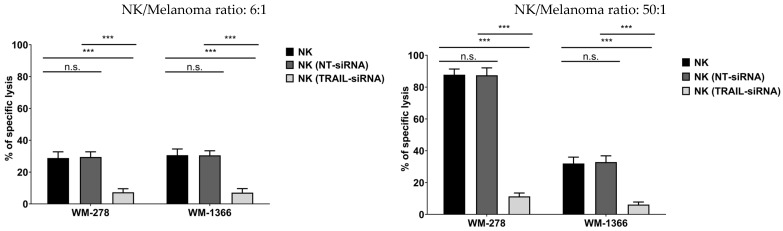
TRAIL silencing inhibits NK cytotoxicity. NK cells were transfected with TRAIL siRNA or non-target siRNA (NT siRNA) before co-culture with melanoma cells at an E:T ratio of 6:1 and 50:1. Cytotoxicity assays were performed in quintuplicates using the calcein release assay. Data are presented as means ± S.E.M. (Student’s *t*-test; *** *p* < 0.001, n.s. not significant).

NK cells kill via two main routes: perforin/granzyme release or death ligand signaling (FASL, TRAIL) [29]. To identify the mechanism of how NK cells kill melanoma cells, NK cells were incubated with an inhibiting anti-TRAIL antibody for 1 h prior to co-culture with melanoma cells; similarly, to investigate the role of the FASL/FAS pathway in NK cell-mediated cytotoxicity, melanoma cells were pre-incubated with an FAS-blocking antibody for 1 h before co-culture with NK cells. To investigate the contribution of granule-dependent NK cell killing, NK cells were incubated with concanavalin (Con A) for 2 h before co-culture with melanoma target cells. The results demonstrate that killing of both the highly NK-sensitive melanoma cell line WM-278 and the low NK-sensitive melanoma cell line WM-1366 is mediated by TRAIL (Figure 2). To confirm the involvement of TRAIL in NK cell-mediated killing of melanoma cells, we performed functional inhibition experiments by transfecting NK cells with specific siRNA to suppress TRAIL expression (Supplementary Figure S3). Following transfection, NK cell cytotoxicity was markedly reduced, providing strong evidence that the TRAIL signaling pathway is a key effector pathway in NK cell-mediated melanoma cell death. These findings underscore the pivotal role of TRAIL in regulating NK cell antitumor activity (Figure 3).

**Figure 4:**
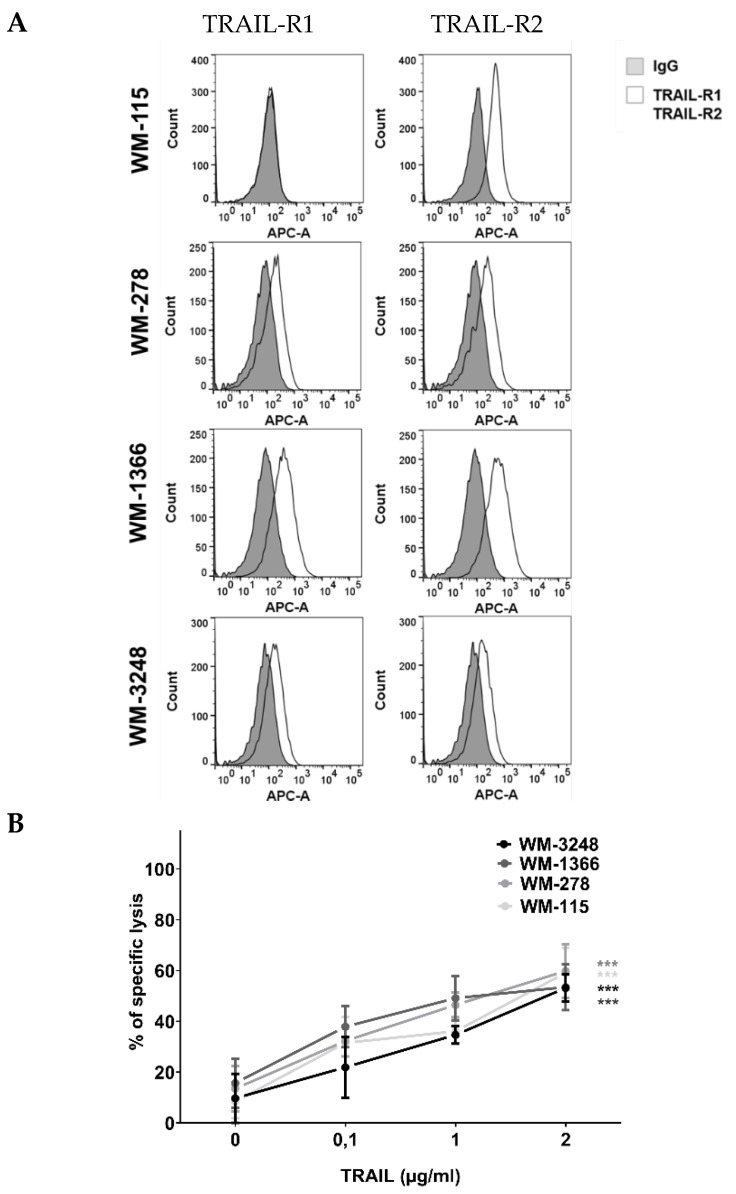
Killing of melanoma cells via TRAIL. (**A**) Surface expression of TRAIL-R1 and TRAIL-R2 in melanoma cells. Data were acquired by flow cytometry and were compared to specific isotype controls. (**B**) Induction of melanoma cell lysis via TRAIL. Cells were exposed to TRAIL (0–2 μg/mL) for 24 h and lysis of melanoma cells was measured via calcein release assay. The 0 µg/mL TRAIL condition is used as the untreated control in this experiment. Data are presented as means ± S.E.M. (Student’s *t*-test; *** *p* < 0.001).

As killing of melanoma cells was predominately mediated by TRAIL, we then investigated whether the TRAIL signaling pathway was impaired in the less sensitive melanoma cell lines WM-1366 and WM-3248. In the first step we examined the expression of TRAIL receptors 1 and 2 by flow cytometry (Figure 4A) and by Western blot (Supplementary Figure S4) in the four cell lines. All four cell lines expressed either one of the two TRAIL receptors.

Since activation of either one of the TRAIL receptors has been previously shown to be sufficient for inducing apoptosis, we next investigated whether recombinant TRAIL was able to induce apoptosis in both highly NK- and low NK-sensitive melanoma cell lines. Therefore, cells were incubated with increasing concentrations of recombinant human TRAIL, and sensitivity to TRAIL was measured using the calcein release assay. All four melanoma cell lines were sensitive to TRAIL-induced killing. At the lowest concentration used, 100 ng/mL, TRAIL killed 21.92% to 37.89% of cells. There were no significant changes in specific lysis in between the four different melanoma cell lines (Figure 4B), excluding differences in sensitivity to TRAIL as a cause for the differential killing of highly NK- and low NK-sensitive melanoma cells. Although NK cell-mediated killing involves TRAIL, the comparable lysis induced by recombinant TRAIL across all cell lines suggests that TRAIL receptor expression is not the limiting factor for NK sensitivity. Therefore, additional mechanisms—inhibitory receptor interactions—are likely to modulate NK cell cytotoxicity.

### 3.3. Role of Negative Checkpoint in NK Cell Killing Against Melanoma

**Figure 5:**
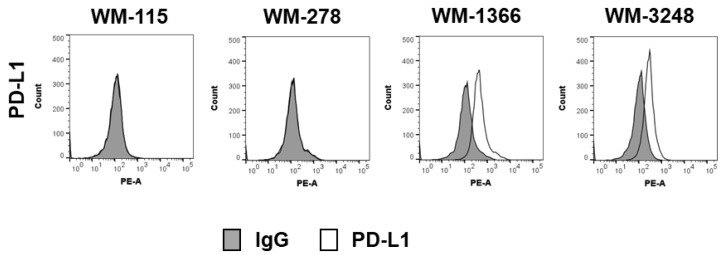
Surface expression of PD-L1 on melanoma cells. PD-L1 expression was analyzed by flow cytometry. Data were compared to specific isotype controls.

As the TRAIL signaling pathway was also functional in the low NK-sensitive melanoma cell lines, we investigated whether inhibition of NK cell killing in cell lines WM-1366 and WM-3248 was due to checkpoint inhibition, of which the PD-L1/PD-1 checkpoint has been shown to be of major clinical relevance in melanoma. We therefore investigated the expression of PD-L1 in the four cell lines. Interestingly, PD-L1 was expressed in both low NK- sensitive cell lines but not in the two highly NK-sensitive cell lines (Figure 5, Supplementary Figure S5).

**Figure 6:**
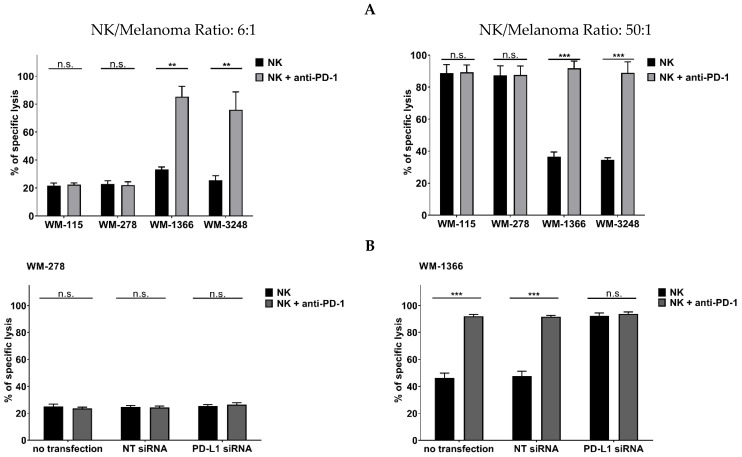
Inhibition of PD-1 increases killing of low NK- sensitive melanoma cells by NK cells. (**A**) NK cells were pretreated with the PD-1 inhibitor nivolumab before co-culturing with melanoma cells at an E:T ratio of 6:1 (**left**) or 50:1 (**right**). (**B**) Melanoma cell lines were transfected with PD-L1 siRNA or non-target siRNA (NT siRNA) before co-culture with NK cells at an E:T ratio of 6:1. Cytotoxicity assays were performed in quintuplicates using the calcein release assay. Data are presented as means ± S.E.M. (Student’s *t*-test; ** *p* < 0.01; *** *p* < 0.001, n.s. not significant).

If expression of PD-L1 by cell lines WM-1366 and WM-3248 is responsible for their low sensitivity against NK cell killing, inhibition of the PD-L1/PD-1 checkpoint by the anti-PD-1 antibody nivolumab should increase their sensitivity to NK cell-mediated killing, as we previously demonstrated that NK cells of healthy persons may express PD-1 to some extent [24,25]. Therefore, NK cells were incubated with nivolumab, before co-culturing them with melanoma cells. Figure 6A shows that preincubation of NK cells with anti-PD-1 antibody markedly increased the sensitivity of melanoma cell lines WM-1366 and WM-3248 to killing by NK cells, whereas nivolumab did not influence the killing of the PD-L1-negative cell lines WM-115 and WM-278. Since nivolumab may activate NK cells via the Fc receptor, we evaluated the drug in the presence of an Fc receptor blocker and results are presented in Supplementary Figure S6. When comparing the results obtained with and without the inhibition of the Fc receptor, no statistically significant differences were observed.

To confirm that the effect of nivolumab on killing was due to inhibition of the PD-L1/PD-1 interaction, PD-L1 expression in melanoma cells was silenced by siRNA. Silencing in melanoma cells WM-1366 and WM-3248 led to downregulation of PD-L1 expression and increased their sensitivity to NK cell-mediated killing (Figure 6B and Supplemental Figure S5).

### 3.4. Role of IFNβ in NK Cell Killing of Melanoma

**Figure 7:**
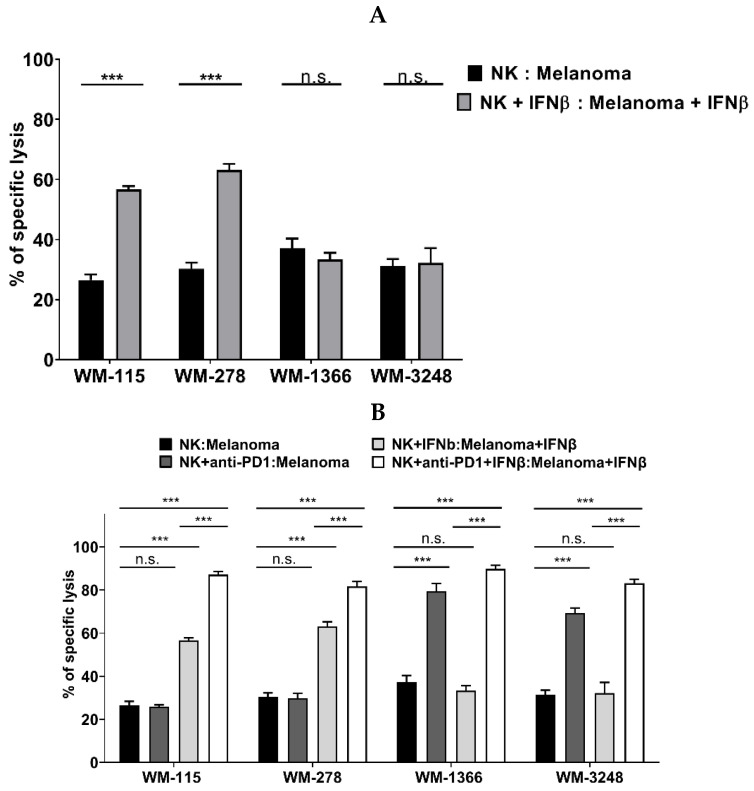
Blocking PD-1 enhances NK-mediated killing of low NK-sensitive melanoma cells in the presence of IFNβ. (**A**) NK cells and melanoma cells were pretreated with IFNβ (1000 U/mL) for 24 h, followed by co-culture at an effector-to-target (E:T) ratio of 6:1. (**B**) IFNβ-activated NK cells were preincubated with the PD-1 inhibitor nivolumab before co-culture with melanoma cells pretreated with IFNβ (1000 U/mL). NK cells were co-cultured with melanoma cell lines at an E:T ratio of 6:1. Cytotoxicity was assessed in quintuplicate using the calcein release assay. Data are shown as mean ± S.E.M. (Student’s *t*-test; *** *p* < 0.001, n.s. not significant).

It was previously shown that NK cells can be activated by type I IFNs, like IFNα and IFNβ [24,26,30]. Therefore, we asked whether the incubation of NK cells and melanoma cells with IFNβ could increase the killing of melanoma cells by NK cells, especially in the cell lines WM-115 and WM-278, which did not profit from PD-L1/PD-1-checkpoint blockade. Incubation of cells with IFNβ significantly increased killing of highly NK- sensitive melanoma cells WM-115 and WM-278 by NK cells. In contrast, killing of low NK- sensitive melanoma cell lines WM-1366 and WM-3248 was either not affected or significantly reduced at E:T ratios of 6:1 and 50:1 (Figure 7A). IFNβ has been shown to upregulate PD-L1 on tumor cells (Supplementary Figure S7A) and PD-1 on NK cells (Supplementary Figure S7B), which we could confirm in our cell lines. We therefore added the anti-PD-1 antibody to IFNβ-pretreated NK and melanoma cells. Whereas nivolumab had no effect in cells WM-115 and WM-278 not pretreated with IFNβ, killing was significantly further enhanced in the presence of IFNβ (Figure 7B), underlining that the sensitization of IFNβ to killing by NK cells could be further augmented by inhibition of the counter effect of IFNβ on the upregulation of the PD-L1/PD-1 checkpoint.

## 4. Discussion

Here, we demonstrate that (1) NK cells kill melanoma cells with varying efficacy, (2) the killing is predominately mediated via the TRAIL signaling pathway, (3) the expression of PD-L1 by melanoma cell lines lowers their sensitivity for NK cell killing and can be reversed by the inhibition of the PD-L1/PD-1 checkpoint, and (4) IFNβ increases the killing of PD-L1-negative cell lines by NK cells, which can be further augmented by blocking the PD-L1/PD-1 checkpoint induced by IFNβ. Our results point to a potential clinical benefit of a combination therapy of IFNβ and PD-L1/PD-1 checkpoint blockade in patients with melanoma.

Melanoma is a highly immunogenic tumor which is composed of tumor cells and tumor-infiltrating lymphocytes. The latter are a polymorphic group of different cellular subsets such as mainly effector T lymphocytes, regulatory T lymphocytes, natural killer cells, dendritic cells, and macrophages [31]. Their distribution and density as well as their activation state can be variable and modulate the clinical outcome [32]. In contrast to complex events required for T cell activation, NK cell activation is governed by the interaction of NK receptors with target cells, independently of antigen processing and presentation. This is one of the reasons why NK cells have gained significant attention in the field of cancer immunotherapy [33]. NK cell cytotoxicity depends on the balance of activating and inhibitory signals on tumor cells, which differ widely in their visibility to NK surveillance and can acquire escape mechanisms. At a 50:1 E:T ratio, WM-278 and WM-115 were highly NK-sensitive, whereas WM-1366 and WM-3248 showed only ~25% lysis, which did not further decrease at 6:1, indicating an early plateau. This plateau likely reflects preferential elimination of NK-sensitive, PD-L1-low cells and enrichment of PD-L1-high, NK-resistant subsets in WM-1366 and WM-3248, where PD-L1-mediated inhibition, rather than effector cell number, limits further NK activity [34,35,36].

**Figure 8:**
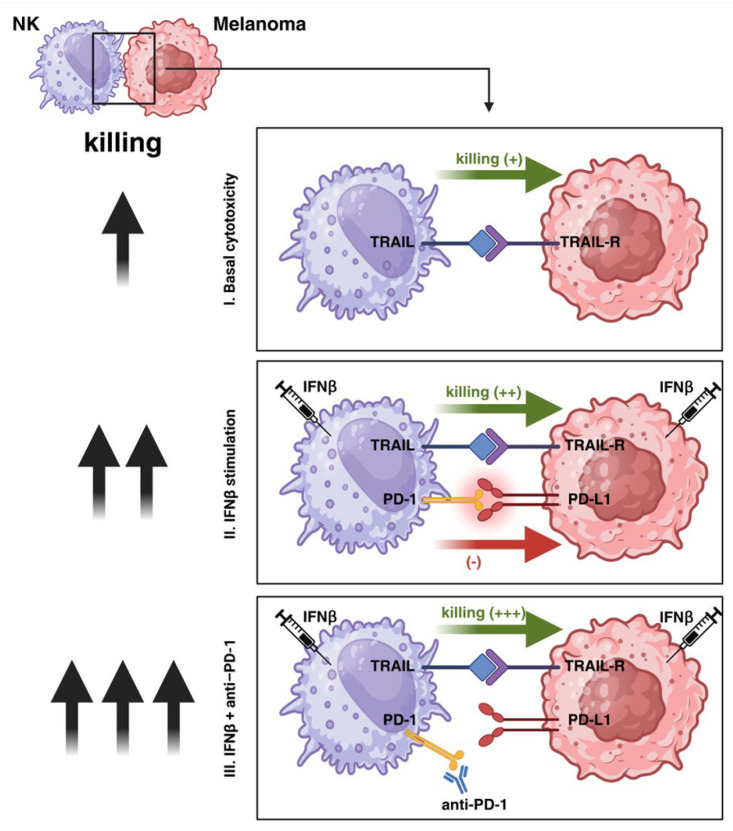
Mechanism of NK cell-mediated killing of melanoma cells. Activation of NK cells by IFNβ enhances their cytotoxic activity against melanoma cells, which is mainly mediated via TRAIL. IFNβ induces (WM-115, WM-278) or upregulates (WM-1366, WM-3248) PD-L1 expression on melanoma cells and PD-1 expression on NK cells. PD-L1 can engage PD-1 receptors on NK cells and subsequently reduce NK cell cytotoxicity (red arrow). Blockade of the PD-1/PD-L1 checkpoint using an anti-PD-1 antibody (nivolumab) restores and further enhances NK cell-mediated cytotoxicity (green arrow). The black arrows on the left represent the level of NK cell cytotoxic activity against melanoma cells. Created with BioRender.com.

By utilizing NK cells derived from healthy donors, we demonstrated their capacity to induce target cell death via the TRAIL pathway in melanoma. TRAIL preferentially induces apoptosis in tumor cells due to differences in receptor expression and downstream signaling compared to non-transformed cells; however, it is not exclusively selective for malignant cells. Under certain conditions, TRAIL can also affect normal tissues, and reports of cytotoxicity in non-tumor contexts exist [37]. However, TRAIL has the characteristic of being cytotoxic only to melanoma cells and not to healthy tissue, including resident melanocytes [38]. As the majority of human melanoma cell lines reported in the literature are sensitive to TRAIL-induced apoptosis [39], our findings postulate a role for NK cells in the tumor defense against melanoma. Investigating the mechanism for the varying sensitivity of melanoma cell lines to NK cell-mediated killing, we could exclude differences in the TRAIL signaling pathway between highly NK- and low NK-sensitive melanoma cell lines. All cell lines expressed TRAIL receptors; they all had a similar sensitivity to apoptosis when incubated with recombinant human TRAIL, ruling out differences downstream in the TRAIL signaling pathway such as over-expression of FADD or decreased expression of caspase-8. We, therefore, focused on differences in the activation status of NK cells when cells were either co-cultured with low NK- and highly NK-sensitive melanoma cell lines. Although melanoma is characterized by tumor-infiltrating lymphocytes, such cells are mostly unable to efficiently control tumor growth in vivo [40,41]. One of the major mechanisms of blocking the activity of immune effector cells is the activation of negative immune checkpoints. Melanoma cells have been shown to express negative checkpoint regulators, such as PD-L1 and CTLA-4 [20]. Interestingly, in contrast to highly NK- sensitive melanoma cell lines, both low NK- sensitive melanoma cell lines expressed PD-L1, and addition of an anti-PD-1 antibody significantly increased their killing by NK cells. In highly NK- sensitive melanoma cell lines, expression of PD-L1 was induced by treatment with IFNβ. In many tumor models, IFNβ concurrently promotes NK cell activation and increases the expression of activating ligands on target cells, effects that can compensate for or override PD-L1-mediated inhibition [24,42,43]. This suggested why, in our overall dataset, IFNβ treatment does not impair NK cell cytotoxicity despite measurable PD-L1 upregulation. In contrast, the melanoma cell lines WM-1366 and WM-3248 represent a distinct biological context. Both cell lines may exhibit a PD-L1-associated phenotype that confers relative resistance to NK-mediated cytotoxicity, as suggested by the observed plateau in lysis even at high E:T ratios. In these cells, IFNβ appears to further enhance PD-L1 expression, and the resulting reinforcement of PD-1/PD-L1 inhibitory signaling could represent a dominant regulatory mechanism. Under such conditions, the suppressive influence of PD-L1 may outweigh the potential sensitizing effect of IFNβ-induced TRAIL receptor upregulation, which might explain the lack of increased NK-mediated killing observed in WM-1366 and WM-3248 (Figure 8).

Addition of the anti-PD-1 antibody nivolumab also significantly increased the cytotoxicity of NK cells against IFNβ-pretreated highly NK- sensitive melanoma cells. Treatment of patients with advanced melanoma with an anti-PD-1-antibody led to a marked increase in survival and resulted in a replacement of interferon-alpha by checkpoint inhibitors [44]. As shown with our experiments, anti-PD-1 antibody and IFNβ complement each other in increasing the activity of NK cells. Similarly, studies in mice demonstrated that addition of anti-PD-1 antibody together with an activator of the type I IFN system-poly (I:C) prolonged survival of mice with melanoma tumors [45]. As type I IFNs activate NK cells on one hand and limit their efficacy on tumor cells on the other hand, by increasing the expression of PD-1 on NK cells and PD-L1 on tumor cells, the combined application of these two agents could increase anti-tumor efficacy. This hypothesis is supported by a phase 2 trial in patients with refractory melanoma which showed a higher response rate for a combination of IFNα and an anti-PD-1 antibody compared to only one substance [46].

## 5. Conclusions

In summary our results show that melanoma cells vary in their sensitivity to NK cell killing due to differences in expression of PD-L1, that activation of the IFNβ system leads to subsequent functional activation of the PD-L1/PD-1 immune-inhibitory signaling axis, and that melanoma cells are most sensitive to killing by NK cells in the presence of IFNβ and anti-PD-1 antibody. These insights provide a rationale for the further development of immune-based therapy protocols for patients with malignant melanoma.

## Data Availability

The original contributions presented in this study are included in the article/Supplementary Material. Further inquiries can be directed to the corresponding author.

## Supplementary Materials

The following supporting information can be downloaded at https://www.mdpi.com/article/10.3390/cancers17243899/s1, Figure S1: Spontaneous calcein release in melanoma cells.; Figure S2: Expression of activated caspase-3 and Ki-67 in melanoma cells.; Figure S3: Surface expression of TRAIL in NK cells.; Figure S4: Expression of TRAIL receptors in melanoma cells; Figure S5: Expression of PD-L1 in melanoma cells.; Figure S6: Comparison of NK cell-mediated lysis of melanoma cell lines with and without anti-PD-1 treatment and Fc receptor silencing.; Figure S7: Expression of PD-L1 on melanoma cells and PD-1 on NK cells after IFNβ treatment.
