# Supplementary material for: Inhibition of PD-L1/PD-1 Checkpoint Increases NK Cell-Mediated Killing of Melanoma Cells in the Presence of Interferon-Beta
Source: Cancers (Basel). 2025 Dec 5;17(24):3899. doi: 10.3390/cancers17243899 (PMC12730462; doi:10.3390/cancers17243899)
Supplement: Supplementary file 1 [file cancers-17-03899-s001.zip › cancers-3915477-supplementary.pdf]

cancers

MDPI

Inhibition of PD-L1/PD-1 Checkpoint Increases NK Cell-Mediated Killing of Melanoma Cells in the Presence of Interferon-Beta

Anna Makowska [1,*], Lian Shen [1], Christina Nothbaum [1], Diana Panayotova-Dimitrova [2], Maria Feoktistova [2], Amir S. Yazdi [2,3] and Udo Kontny [1,3]

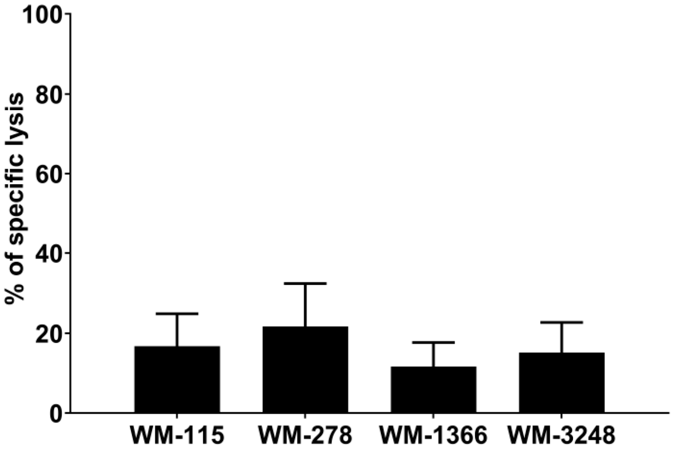

Supplemental Figure S1. Spontaneous Calcein Release in Melanoma Cells. Spontaneous release of calcein (cell lysis) was assessed in melanoma cell cultures without additional stimuli. Cells were incubated with 15 µM calcein-acetoxymethyl ester (calcein-AM) for 30 minutes at 37 °C to allow intracellular dye loading. Following the loading period, cells were washed to remove excess dye and subsequently cultured under standard conditions for 4 hours. After incubation, the release of calcein into the supernatant was quantified using a SpectraMax microplate reader, measuring fluorescence intensity as an indicator of membrane integrity and passive dye efflux. This result provides a baseline measurement of spontaneous melanoma lysis cells under physiological conditions.

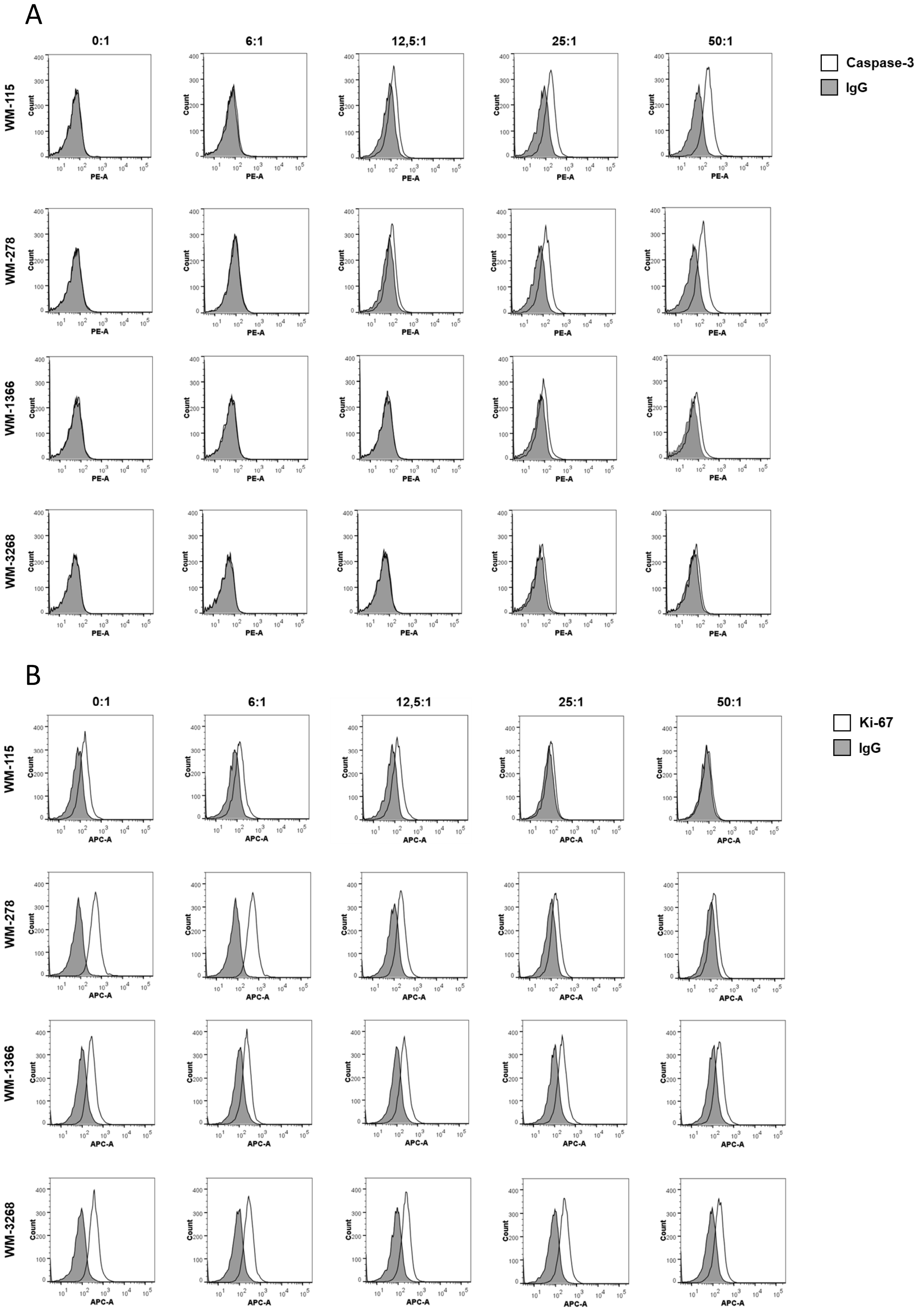

Supplemental Figure S2. Expression of activated Caspase-3 and Ki-67 in melanoma cells. Melanoma cells were co-cultured with NK cells at effector-to-target (E:T) ratios ranging from 1:1 to 50:1. After 4 hours of co-culture, caspase-3 (A) and Ki-67 (B) expression was measured by flow cytometry and compared with the corresponding isotype controls.

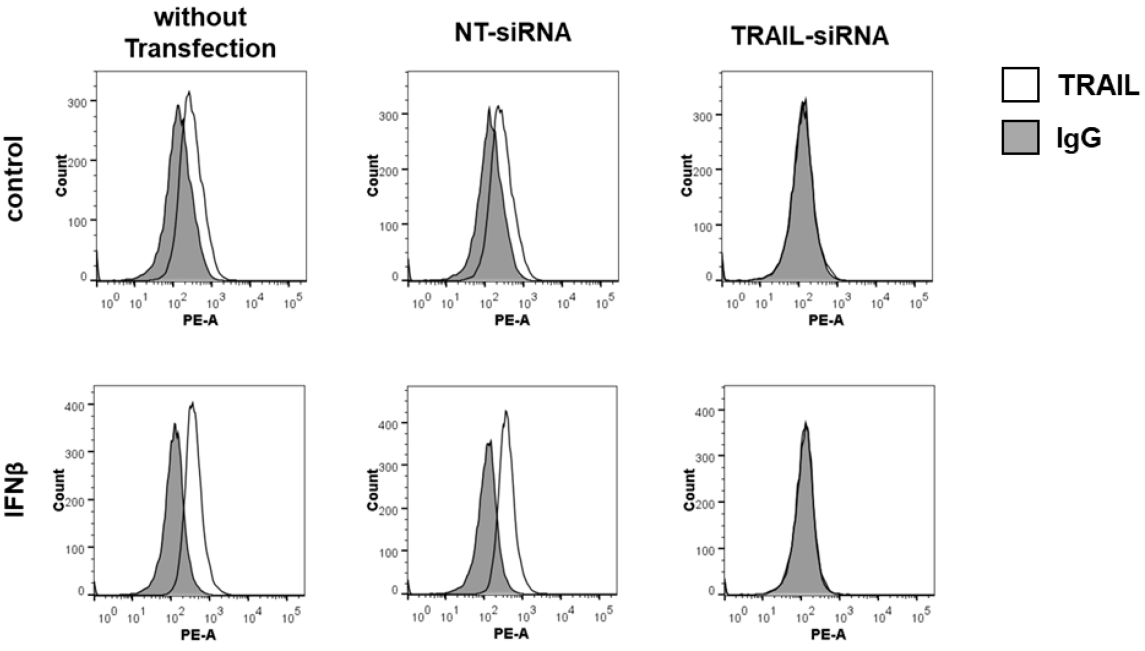

Supplemental Figure S3. Surface expression of TRAIL in NK cells. NK cells were transfected with TRAIL siRNA for 16 h and then incubated with IFNβ at 1,000 U / ml for 24 h. Expression of TRAIL was analyzed flow cytometry and compared with the corresponding isotype controls.

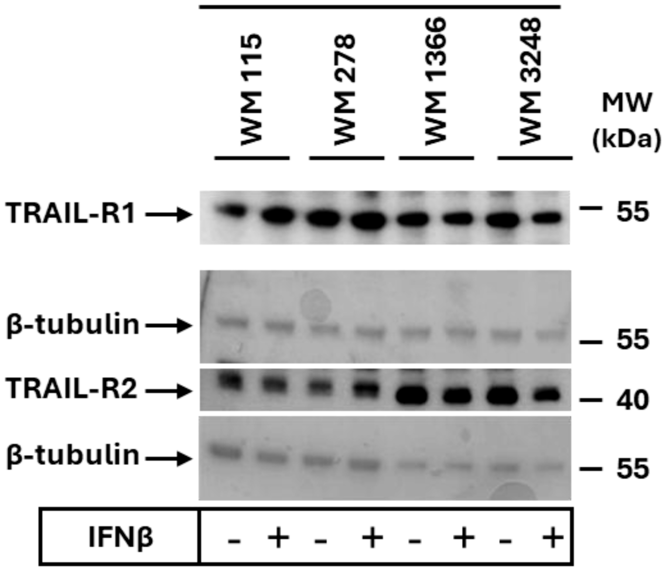

Supplemental Figure S4. Surface expression of TRAIL receptors in melanoma cells. Melanoma cells were incubated with IFNβ at 1,000 U / ml for 24 h. Expression of TRAIL-R1 and TRAIL-R2 was analyzed by immunoblot. Beta tubulin was used as loading control.

A

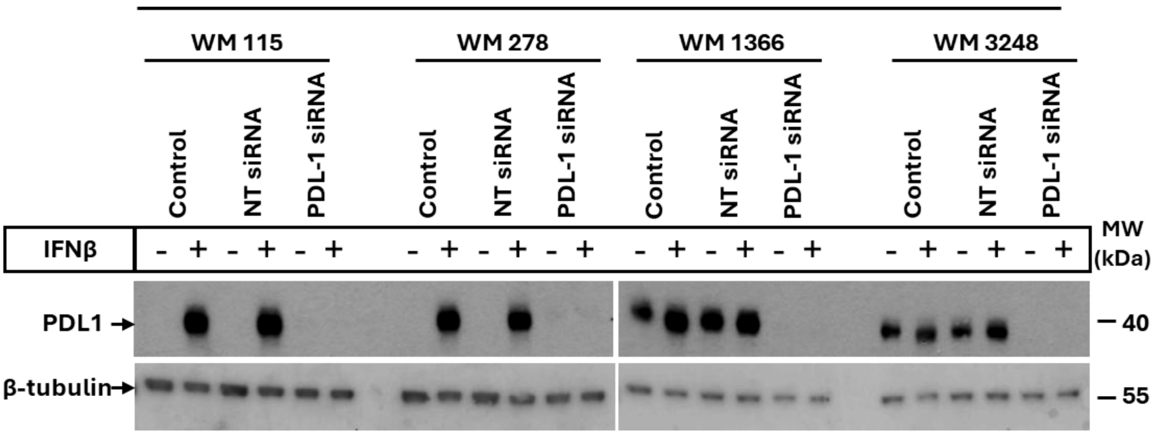

B

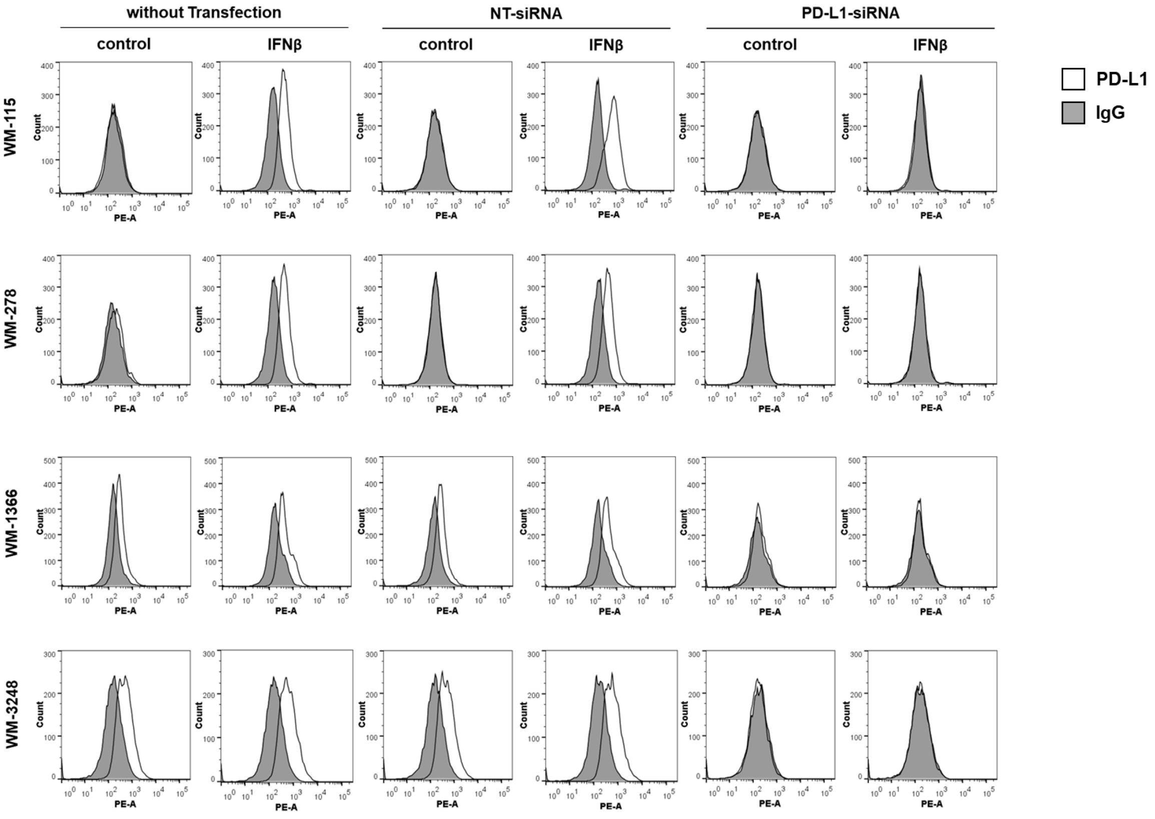

Supplemental Figure S5. PD-L1 expression in melanoma cells. Melanoma cells were transfected with PD-L1–specific siRNA for 16 h, followed by stimulation with IFNβ (1,000 U/mL) for 24 h. PD-L1 expression was assessed by immunoblotting, with β-tubulin serving as a loading control (A). Surface PD-L1 expression was further evaluated by flow cytometry and compared to the respective isotype controls (B).

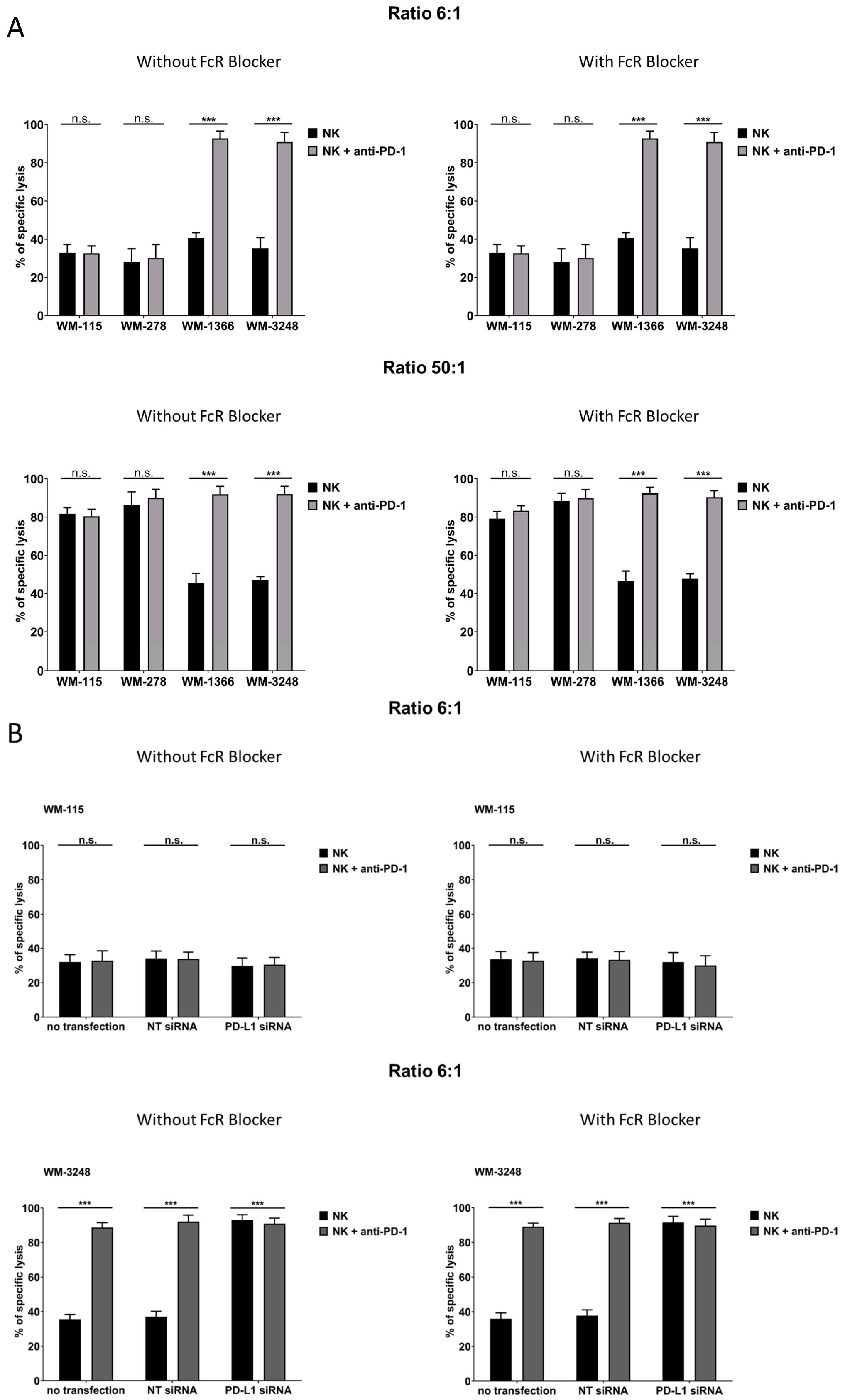

Supplemental Figure S6. Comparison of NK cell-mediated lysis of melanoma cell lines with and without anti-PD-1 treatment and Fc receptor silencing. Two experimental panels were performed: (1) without Fc receptor blocker; (2) with Fc receptor blocker (NK cells were treated with an Fc receptor blocker 1 h before nivolumab administration).(A) NK cells were pretreated with the anti-PD-1 antibody nivolumab before co-culturing with melanoma cells at E:T ratios of 6:1 and 50:1. (B) Melanoma cell lines were transfected with PD-L1 siRNA or non-targeting siRNA (NT siRNA) before

co-culture with NK cells at an E:T ratio of 6:1. Cytotoxicity assays were performed in quintuplicate using the calcein release assay. Data are presented as means ± S.E.M. (Student's t-test; * $p < 0.05$; ** $p < 0.01$; *** $p < 0.001$).

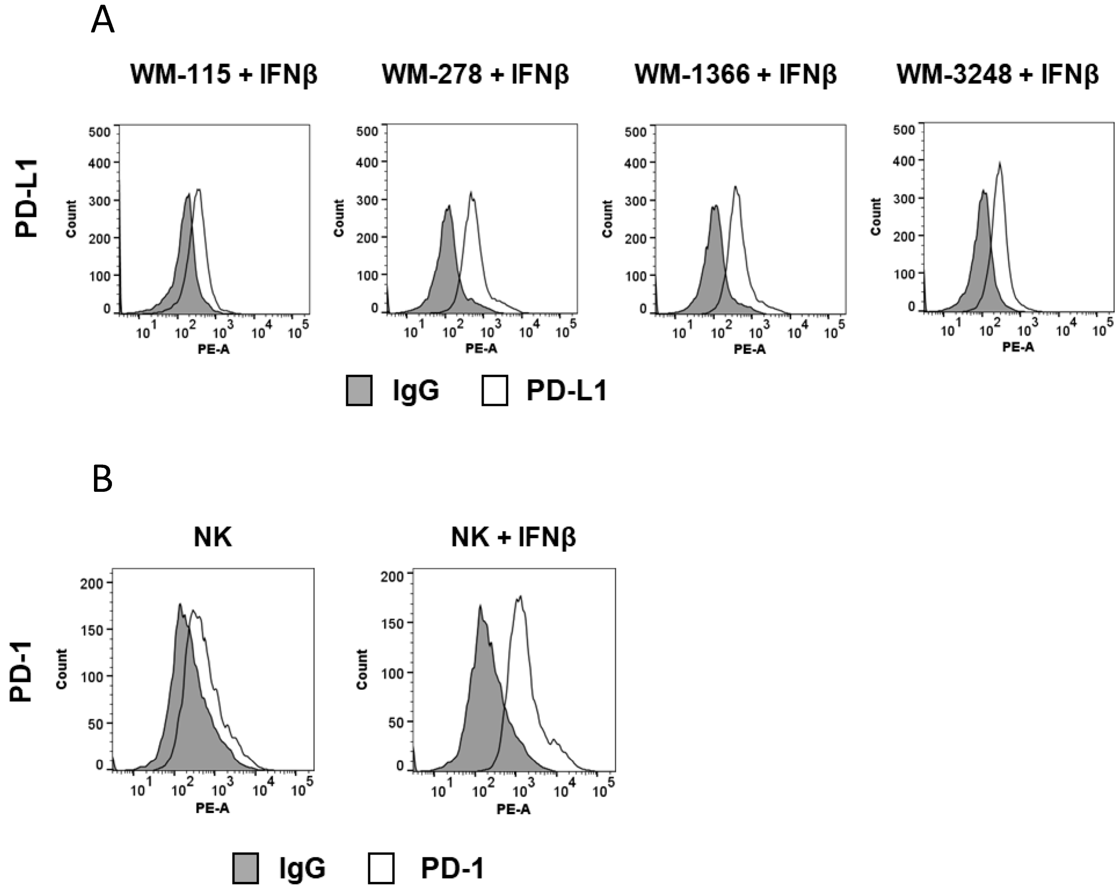

Supplemental Figure S7. Expression of PD-L1 on melanoma cells and PD-1 on NK-cells after IFNβ treatment. Cells were pretreated with 1,000 U / ml IFNβ for 24 h. PD-L1 (Melanoma cells -A)- and PD-1 (NK cells -B) expression was analyzed by flow cytometry. Data were compared to specific isotype controls.
